# Supplementary material for: Collectively enhanced Ramsey readout by cavity sub- to superradiant transition
Source: Nat Commun. 2024 Feb 5;15:1084. doi: 10.1038/s41467-024-45420-x (PMC10844618; doi:10.1038/s41467-024-45420-x)
Supplement: Supplementary file 1 — Supplementary Information [file 41467_2024_45420_MOESM1_ESM.pdf]

Supplementary information: Collectively enhanced Ramsey readout by cavity sub- to superradiant transition

Eliot Bohr[1*†], Sofus L. Kristensen[1†], Christoph Hotter[2], Stefan Alaric Schäffer[1], Julian Robinson-Tait[1], Jan W. Thomsen[1], Tanya Zelevinsky[3], Helmut Ritsch[2], Jörg Helge Müller[1]

[1]Niels Bohr Institute, University of Copenhagen, Blegdamsvej 17, Copenhagen, DK-2100, Denmark.
[2]Institut für Theoretische Physik, Universität Innsbruck, Technikerstr. 21a, Innsbruck, A-6020, Austria.
[3]Department of Physics, Columbia University, 538 West 120th Street, New York, 10027-5255, NY, USA.

*Corresponding author(s). E-mail(s): eliot.bohr@nbi.ku.dk;
†These authors contributed equally to this work.

1 Supplementary Note 1

In the following, we provide details on the numerical simulations to create the theoretical curves in Fig. 2b, Fig. 2c and Fig. 3 of the main article. The fundamental theoretical model and a thorough interpretation of the physical phenomena can be found in [1]. The simulations have been performed with the open-source Julia package QuantumCumulants.jl [2]. The supplementary information additionally includes two code examples: **cavity_sub-to-superradiance_simulations** to reproduce the simulation results and **cavity_sub-to-superradiance_equations** to show the generic second-order cumulant equations for our system.

1.1 Details of the model

To model the experimental system we consider N two-level atoms coupled to a single-mode cavity. The atoms are coherently driven, described by a time-dependent Rabi frequency $\Omega(t)$. The Hamiltonian of this system in the rotating frame of the drive laser

is [1]

$$H = -\delta_{\mathrm{c}} a^\dagger a + \sum_{j=1}^{N} \Big[- \delta_{\mathrm{a}} \sigma_j^{22} + g_j (a^\dagger \sigma_j^{12} + a \sigma_j^{21}) + \frac{\Omega(t)}{2} (\sigma_j^{21} + \sigma_j^{12}) \Big], \tag{1}$$

with the cavity photon creation (annihilation) operator $a^\dagger$ (a) and the atomic transition operator $\sigma_j^{kl} = |k\rangle_j \langle l|_j$ for the j-th atom. In Eq. (1), $\delta_{\mathrm{a}} = \omega_{\mathrm{l}} - \omega_{\mathrm{a}}$ is the detuning between the drive laser and the atomic transition frequency, $\delta_{\mathrm{c}} = \omega_{\mathrm{l}} - \omega_{\mathrm{c}}$ describes the detuning between the laser and the cavity resonance frequency and g_j is the atom-cavity coupling of the j-th atom.

The dissipative processes, namely cavity photon decay and individual atomic decay, are described by the Liouvillian $\mathcal{L}[\rho]$ in the master equation $\dot{\rho} = i\,[\rho, H] + \mathcal{L}\,[\rho]$. In the Born-Markov approximation [3] we can write the Liouvillian in Lindblad form as

$$\mathcal{L}\,[\rho] = \sum_i R_i \left(2 J_i \rho J_i^\dagger - J_i^\dagger J_i \rho - \rho J_i^\dagger J_i \right), \tag{2}$$

with the jump operators $\{J_i\}$ and their corresponding rates $\{R_i\}$. Cavity photon losses are described with the jump operator a and corresponding rate κ, the individual atomic decay of the j-th atom is described by the jump operator σ_j^{12} and corresponding atomic decay rate γ.

To simulate the large open quantum system, involving several million atoms and a cavity mode, we use a second-order cumulant expansion [2, 4]. Furthermore, we approximate the atomic positions in the following way: First, since the temperature of the atomic cloud is around $2\,\mu$K, we assume the atomic position to be stationary for the duration of the experiment. Moreover, we sample the atomic distribution along the cavity axis with eight different equally populated positions along the sine-shaped cavity mode function. The explicit eight positions x_j, corresponding to a coupling $g_j = g \sin(2\pi x_j/\lambda)$, are spaced equidistant at $x_j = [\pm 1/8, \pm 3/8, \pm 5/8, \pm 7/8]\lambda/4$. The distribution of the atomic cloud perpendicular to the cavity axis is not modeled, due to its relatively small extension compared to the cavity mode volume. Note that we consider the same laser excitation phase for all atoms. In principle, atoms located along the drive laser axis will pick up different phases. However, the physically relevant feature, namely an overall vanishing relative phase, is already incorporated with the positive and negative cavity coupling [1]. This makes sampling of the drive laser phase unnecessary. The drive laser intensity is assumed to be equal for all atoms since the laser beam waist is much larger than the atomic cloud. Direct atom-atom interaction is not included. For the initial state of the system, we assume all atoms in the ground state and no photons in the cavity.

The general second-order equations (without sampling the positions) are derived with QuantumCumulants.jl [2] and are shown in the file **cavity_sub-to-superradiance_equations**.

1.2 Numerical parameters

The natural frequency unit equal to one in our simulations is 1 kHz, meaning e.g. that the spontaneous emission rate of $2\pi \times 7.5$ kHz is described by $\gamma = 2\pi \times 7.5$ in the numerical simulation. For all simulations we use $\gamma = 2\pi \times 7.5$ kHz, $\kappa = 2\pi \times 780$ kHz and $\Omega = 2\pi \times 833$ kHz (corresponding to a $\pi/2$-pulse duration of 300 ns). For the atom-cavity coupling, we use an effective maximal strength of $g_0 = 2\pi \times 610$ Hz, corresponding to the normal mode splitting measurement.

For the threshold and delay time results shown in Fig. 2b and Fig. 2c we use $N = 2 \times 10^7$. To simulate the pulsed excitation we use a step function for $\Omega(t)$ which is always 0 except for $0 \leq t \geq t_P$ where it is equal to $\Omega = 2\pi \times 833$ kHz.

For the Ramsey results in Fig. 3 we use $N = 4 \times 10^7$ atoms. The Ramsey pulse sequence is simulated with two rectangular step functions for $\Omega(t)$ with duration $t_P = \pi/2\Omega$ separated (end-to-end) by the free evolution time $T = 5 \times 10^{-6}$ s. In the file **cavity_sub-to-superradiance_simulations** we provide the full code to reproduce the simulation results. Note, that we reduced the resolution of the scans to decrease the computation time.

2 Supplementary Note 2

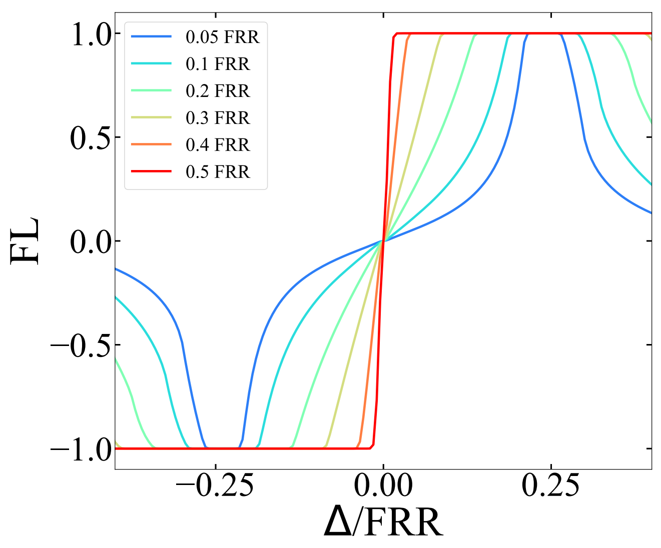

Supplementary Fig. 1 | FL discriminator. Conversion slope for various frequency step sizes. The red curve corresponds to interrogating at the kinks of the collectively enhanced lineshape and leads to a very steep discrimator slope with a very little dynamic range. As we decrease the step size the slope decreases and dynamic range increases.

We define a frequency locator, FL $= \langle \frac{P_{i+1}-P_i}{P_{i+1}+P_i} \rangle$, where P_i is the peak intensity of the ith pulse within a cycle. We also define the frequency spacing of the Ramsey fringes the free Ramsey range (FRR), which is given by the inverse of the interpulsar free evolution time.

In Supplementary Fig. 1 we calculate the discriminator shape from the simulated Ramsey fringes for various frequency step sizes ranging from 0.01 FRR to 0.5 FRR. For a step size of 0.5 FRR, which corresponds to interrogating at the kinks, there is a very steep slope but a very small dynamic range. As we decrease the step size the slope decreases and dynamic range increases.

Optimizing the technique involves choosing frequency steps to obtain a suitable balance between the FL slope and the signal-to-noise ratio (SNR) to form a deterministic measurement of the frequency deviation. With an improved SNR, the frequency steps can be tuned to obtain the steepest slope by probing close to the FL kinks. A realistic possibility would be to incorporate a series of measurements with different step sizes, or different free evolution periods, T.

The new lineshape warrants further investigation of optimal phase/frequency stepping and increasing the SNR of the spectroscopic lineshape before qualified judgment can be made on how to optimally gain the feedback signal.

References

[1] Hotter, C., Ostermann, L., Ritsch, H.: Cavity sub- and superradiance for transversely driven atomic ensembles. Phys. Rev. Res. **5**, 013056 (2023) https://doi.org/10.1103/PhysRevResearch.5.013056

[2] Plankensteiner, D., Hotter, C., Ritsch, H.: QuantumCumulants.jl: A Julia framework for generalized mean-field equations in open quantum systems. Quantum **6**, 617 (2022) https://doi.org/10.22331/q-2022-01-04-617

[3] Gardiner, C., Zoller, P.: Quantum noise: a handbook of Markovian and non-Markovian quantum stochastic methods with applications to quantum optics. Springer (2004)

[4] Kubo, R.: Generalized cumulant expansion method. Journal of the Physical Society of Japan **17**(7), 1100–1120 (1962) https://doi.org/10.1143/JPSJ.17.1100
