# Supplementary material for: Collectively enhanced Ramsey readout by cavity sub- to superradiant transition
Source: Nat Commun. 2024 Feb 5;15:1084. doi: 10.1038/s41467-024-45420-x (PMC10844618; doi:10.1038/s41467-024-45420-x)
Supplement: Supplementary file 3 — Description of Additional Supplementary Files [file 41467_2024_45420_MOESM3_ESM.pdf]

Description of Additional Supplementary Files

File name: Supplementary Code 1

Description: cavity_sub-to-superradiance_equations: Julia program using QuantumCumulants.jl to derive and show the generic second-order cumulant equations for our system.

File name: Supplementary Code 2

Description: cavity_sub-to-superradiance_simulations: Second-order cumulant simulation with the open-source Julia package QuantumCumulants.jl to reproduce the theory results for our system.
