# Supplementary material for: Collectively enhanced Ramsey readout by cavity sub- to superradiant transition
Source: Nat Commun. 2024 Feb 5;15:1084. doi: 10.1038/s41467-024-45420-x (PMC10844618; doi:10.1038/s41467-024-45420-x)
Supplement: Supplementary file 4 — Supplementary Code 1 [file 41467_2024_45420_MOESM4_ESM.html]

cavity\_sub-to-superradiance\_equations


# Cavity Sub- to Superradiance - Equations¶

This program is only used to display the generic system of equations. To solve the system numerically, we scale the atom number. However, with QuantumCumulants this is currently only possible by explicitly defining the (eight) different atom clusters and then scaling them individually. This is shown in the code example **cavity\_sub-to-superradiance\_equations**.

In [1]:

```
#Julia-1.9.1
using QuantumCumulants #v0.2.23
```

In [2]:

```
# Hilbert space
hf = FockSpace(:cavity) 
ha = NLevelSpace(:atom,2)
h = hf ⊗ ha

# Fundamental operators
a = Destroy(h,:a,1) 
σ(i,j,k) = IndexedOperator(Transition(h,:σ,i,j,2), k) 

# Parameters
@cnumbers γ κ δc δa Ω N
g(k) = IndexedVariable(:g, k)

# Summation indices
i = Index(h,:i,N,ha)
k = Index(h,:k,N,ha);
```

In [3]:

```
# Hamiltonian
H_0 = -δc*a'a - δa*( ∑(σ(2,2,k),k))
H_int = (a'∑(g(k)*σ(1,2,k),k) + a*∑(g(k)*σ(2,1,k),k))
H_laser = Ω/2*( ∑(σ(1,2,k),k) + ∑(σ(2,1,k),k)) 
H = H_0 + H_int + H_laser

# Jump operators & rates
J = [a, σ(1,2,k)] 
R = [κ, γ];
```

In [4]:

```
ops = [a'a, σ(2,2,i), a'σ(1,2,i)]
eqs = meanfield(ops, H, J; rates=R, order=2)
```

Out[4]:

\begin{align}
\frac{d}{dt} \langle a^\dagger a\rangle =& 1 i \underset{k}{\overset{N}{\sum}} {g}\_{k} \langle a {\sigma}\_{k}^{{21}}\rangle -1 i \underset{k}{\overset{N}{\sum}} {g}\_{k} \langle a^\dagger {\sigma}\_{k}^{{12}}\rangle -1.0 \langle a^\dagger a\rangle \kappa \\
\frac{d}{dt} \langle {\sigma}\_{i}^{{22}}\rangle =& 1 i {g}\_{i} \langle a^\dagger {\sigma}\_{i}^{{12}}\rangle -1 i {g}\_{i} \langle a {\sigma}\_{i}^{{21}}\rangle + \frac{-1}{2} i \langle {\sigma}\_{i}^{{21}}\rangle \Omega + \frac{1}{2} i \langle {\sigma}\_{i}^{{12}}\rangle \Omega -1.0 \langle {\sigma}\_{i}^{{22}}\rangle \gamma \\
\frac{d}{dt} \langle a^\dagger {\sigma}\_{i}^{{12}}\rangle =& 1 i \underset{k{\ne}i}{\overset{N}{\sum}} {g}\_{k} \langle {\sigma}\_{k}^{{21}} {\sigma}\_{i}^{{12}}\rangle -1 i {g}\_{i} \langle a^\dagger a\rangle + 1 i {g}\_{i} \langle {\sigma}\_{i}^{{22}}\rangle + \frac{-1}{2} i \langle a^\dagger\rangle \Omega -0.5 \langle a^\dagger {\sigma}\_{i}^{{12}}\rangle \left( \gamma + \kappa \right) + 1 i \langle a^\dagger {\sigma}\_{i}^{{12}}\rangle {\delta}a -1 i \langle a^\dagger {\sigma}\_{i}^{{12}}\rangle {\delta}c + 1 i \langle a^\dagger {\sigma}\_{i}^{{22}}\rangle \Omega + 2 i {g}\_{i} \left( \langle a^\dagger\rangle \langle a {\sigma}\_{i}^{{22}}\rangle + \langle a^\dagger a\rangle \langle {\sigma}\_{i}^{{22}}\rangle + \langle a^\dagger {\sigma}\_{i}^{{22}}\rangle \langle a\rangle -2 \langle a^\dagger\rangle \langle a\rangle \langle {\sigma}\_{i}^{{22}}\rangle \right)
\end{align}

In [5]:

```
eqs_c = complete(eqs) # closed set of equations (in 2nd-order)
```

Out[5]:

\begin{align}
\frac{d}{dt} \langle a^\dagger a\rangle =& 1 i \underset{k}{\overset{N}{\sum}} {g}\_{k} \langle a {\sigma}\_{k}^{{21}}\rangle -1 i \underset{k}{\overset{N}{\sum}} {g}\_{k} \langle a^\dagger {\sigma}\_{k}^{{12}}\rangle -1.0 \langle a^\dagger a\rangle \kappa \\
\frac{d}{dt} \langle {\sigma}\_{i}^{{22}}\rangle =& 1 i {g}\_{i} \langle a^\dagger {\sigma}\_{i}^{{12}}\rangle -1 i {g}\_{i} \langle a {\sigma}\_{i}^{{21}}\rangle + \frac{-1}{2} i \langle {\sigma}\_{i}^{{21}}\rangle \Omega + \frac{1}{2} i \langle {\sigma}\_{i}^{{12}}\rangle \Omega -1.0 \langle {\sigma}\_{i}^{{22}}\rangle \gamma \\
\frac{d}{dt} \langle a^\dagger {\sigma}\_{i}^{{12}}\rangle =& 1 i \underset{k{\ne}i}{\overset{N}{\sum}} {g}\_{k} \langle {\sigma}\_{k}^{{21}} {\sigma}\_{i}^{{12}}\rangle -1 i {g}\_{i} \langle a^\dagger a\rangle + 1 i {g}\_{i} \langle {\sigma}\_{i}^{{22}}\rangle + \frac{-1}{2} i \langle a^\dagger\rangle \Omega -0.5 \langle a^\dagger {\sigma}\_{i}^{{12}}\rangle \left( \gamma + \kappa \right) + 1 i \langle a^\dagger {\sigma}\_{i}^{{12}}\rangle {\delta}a -1 i \langle a^\dagger {\sigma}\_{i}^{{12}}\rangle {\delta}c + 1 i \langle a^\dagger {\sigma}\_{i}^{{22}}\rangle \Omega + 2 i {g}\_{i} \left( \langle a^\dagger\rangle \langle a {\sigma}\_{i}^{{22}}\rangle + \langle a^\dagger a\rangle \langle {\sigma}\_{i}^{{22}}\rangle + \langle a^\dagger {\sigma}\_{i}^{{22}}\rangle \langle a\rangle -2 \langle a^\dagger\rangle \langle a\rangle \langle {\sigma}\_{i}^{{22}}\rangle \right) \\
\frac{d}{dt} \langle {\sigma}\_{i}^{{21}}\rangle =& \frac{1}{2} i \Omega + 1 i {g}\_{i} \langle a^\dagger\rangle -2 i {g}\_{i} \langle a^\dagger {\sigma}\_{i}^{{22}}\rangle -0.5 \langle {\sigma}\_{i}^{{21}}\rangle \gamma -1 i \langle {\sigma}\_{i}^{{21}}\rangle {\delta}a -1 i \langle {\sigma}\_{i}^{{22}}\rangle \Omega \\
\frac{d}{dt} \langle a^\dagger\rangle =& 1 i \underset{k}{\overset{N}{\sum}} {g}\_{k} \langle {\sigma}\_{k}^{{21}}\rangle -1 i \langle a^\dagger\rangle {\delta}c -0.5 \langle a^\dagger\rangle \kappa \\
\frac{d}{dt} \langle a^\dagger {\sigma}\_{i}^{{22}}\rangle =& 1 i \underset{k{\ne}i}{\overset{N}{\sum}} {g}\_{k} \langle {\sigma}\_{k}^{{21}} {\sigma}\_{i}^{{22}}\rangle + \frac{-1}{2} i \langle a^\dagger {\sigma}\_{i}^{{21}}\rangle \Omega + \frac{1}{2} i \langle a^\dagger {\sigma}\_{i}^{{12}}\rangle \Omega -1.0 \langle a^\dagger {\sigma}\_{i}^{{22}}\rangle \gamma -1 i \langle a^\dagger {\sigma}\_{i}^{{22}}\rangle {\delta}c -0.5 \langle a^\dagger {\sigma}\_{i}^{{22}}\rangle \kappa + 1 i {g}\_{i} \left( \langle a^\dagger a^\dagger\rangle \langle {\sigma}\_{i}^{{12}}\rangle + 2 \langle a^\dagger\rangle \langle a^\dagger {\sigma}\_{i}^{{12}}\rangle -2 \langle a^\dagger\rangle ^{2} \langle {\sigma}\_{i}^{{12}}\rangle \right) -1 i {g}\_{i} \left( \langle a^\dagger {\sigma}\_{i}^{{21}}\rangle \langle a\rangle + \langle a^\dagger\rangle \langle a {\sigma}\_{i}^{{21}}\rangle + \langle {\sigma}\_{i}^{{21}}\rangle \langle a^\dagger a\rangle -2 \langle a^\dagger\rangle \langle {\sigma}\_{i}^{{21}}\rangle \langle a\rangle \right) \\
\frac{d}{dt} \langle {\sigma}\_{i}^{{12}} {\sigma}\_{j}^{{21}}\rangle =& -1 i \langle a {\sigma}\_{j}^{{21}}\rangle {g}\_{i} + 1 i \langle a^\dagger {\sigma}\_{i}^{{12}}\rangle {g}\_{j} -1.0 \langle {\sigma}\_{i}^{{12}} {\sigma}\_{j}^{{21}}\rangle \gamma -1 i \langle {\sigma}\_{i}^{{12}} {\sigma}\_{j}^{{22}}\rangle \Omega + \frac{1}{2} i \langle {\sigma}\_{i}^{{12}}\rangle \Omega + \frac{-1}{2} i \langle {\sigma}\_{j}^{{21}}\rangle \Omega + 1 i \langle {\sigma}\_{i}^{{22}} {\sigma}\_{j}^{{21}}\rangle \Omega -2 i \left( \langle a^\dagger {\sigma}\_{i}^{{12}}\rangle \langle {\sigma}\_{j}^{{22}}\rangle + \langle a^\dagger {\sigma}\_{j}^{{22}}\rangle \langle {\sigma}\_{i}^{{12}}\rangle + \langle a^\dagger\rangle \langle {\sigma}\_{i}^{{12}} {\sigma}\_{j}^{{22}}\rangle -2 \langle a^\dagger\rangle \langle {\sigma}\_{i}^{{12}}\rangle \langle {\sigma}\_{j}^{{22}}\rangle \right) {g}\_{j} + 2 i \left( \langle a {\sigma}\_{j}^{{21}}\rangle \langle {\sigma}\_{i}^{{22}}\rangle + \langle a {\sigma}\_{i}^{{22}}\rangle \langle {\sigma}\_{j}^{{21}}\rangle + \langle a\rangle \langle {\sigma}\_{i}^{{22}} {\sigma}\_{j}^{{21}}\rangle -2 \langle a\rangle \langle {\sigma}\_{j}^{{21}}\rangle \langle {\sigma}\_{i}^{{22}}\rangle \right) {g}\_{i} \\
\frac{d}{dt} \langle a^\dagger {\sigma}\_{i}^{{21}}\rangle =& 1 i \underset{k{\ne}i}{\overset{N}{\sum}} {g}\_{k} \langle {\sigma}\_{k}^{{21}} {\sigma}\_{i}^{{21}}\rangle + 1 i {g}\_{i} \langle a^\dagger a^\dagger\rangle -0.5 \langle a^\dagger {\sigma}\_{i}^{{21}}\rangle \left( \gamma + \kappa \right) -1 i \langle a^\dagger {\sigma}\_{i}^{{21}}\rangle \left( {\delta}a + {\delta}c \right) + \frac{1}{2} i \langle a^\dagger\rangle \Omega -1 i \langle a^\dagger {\sigma}\_{i}^{{22}}\rangle \Omega -2 i {g}\_{i} \left( \langle a^\dagger a^\dagger\rangle \langle {\sigma}\_{i}^{{22}}\rangle + 2 \langle a^\dagger\rangle \langle a^\dagger {\sigma}\_{i}^{{22}}\rangle -2 \langle a^\dagger\rangle ^{2} \langle {\sigma}\_{i}^{{22}}\rangle \right) \\
\frac{d}{dt} \langle a^\dagger a^\dagger\rangle =& 2 i \underset{k}{\overset{N}{\sum}} {g}\_{k} \langle a^\dagger {\sigma}\_{k}^{{21}}\rangle -2 i \langle a^\dagger a^\dagger\rangle {\delta}c -1.0 \langle a^\dagger a^\dagger\rangle \kappa \\
\frac{d}{dt} \langle {\sigma}\_{i}^{{12}} {\sigma}\_{j}^{{22}}\rangle =& -1 i \langle a {\sigma}\_{j}^{{22}}\rangle {g}\_{i} + \frac{1}{2} i \langle {\sigma}\_{i}^{{12}} {\sigma}\_{j}^{{12}}\rangle \Omega + \frac{-1}{2} i \langle {\sigma}\_{i}^{{12}} {\sigma}\_{j}^{{21}}\rangle \Omega -1.5 \langle {\sigma}\_{i}^{{12}} {\sigma}\_{j}^{{22}}\rangle \gamma + 1 i \langle {\sigma}\_{i}^{{12}} {\sigma}\_{j}^{{22}}\rangle {\delta}a + 1 i \langle {\sigma}\_{i}^{{22}} {\sigma}\_{j}^{{22}}\rangle \Omega + \frac{-1}{2} i \langle {\sigma}\_{j}^{{22}}\rangle \Omega + 1 i \left( \langle a^\dagger {\sigma}\_{i}^{{12}}\rangle \langle {\sigma}\_{j}^{{12}}\rangle + \langle a^\dagger {\sigma}\_{j}^{{12}}\rangle \langle {\sigma}\_{i}^{{12}}\rangle + \langle a^\dagger\rangle \langle {\sigma}\_{i}^{{12}} {\sigma}\_{j}^{{12}}\rangle -2 \langle a^\dagger\rangle \langle {\sigma}\_{i}^{{12}}\rangle \langle {\sigma}\_{j}^{{12}}\rangle \right) {g}\_{j} -1 i \left( \langle a {\sigma}\_{i}^{{12}}\rangle \langle {\sigma}\_{j}^{{21}}\rangle + \langle a {\sigma}\_{j}^{{21}}\rangle \langle {\sigma}\_{i}^{{12}}\rangle + \langle a\rangle \langle {\sigma}\_{i}^{{12}} {\sigma}\_{j}^{{21}}\rangle -2 \langle a\rangle \langle {\sigma}\_{i}^{{12}}\rangle \langle {\sigma}\_{j}^{{21}}\rangle \right) {g}\_{j} + 2 i \left( \langle a {\sigma}\_{i}^{{22}}\rangle \langle {\sigma}\_{j}^{{22}}\rangle + \langle a {\sigma}\_{j}^{{22}}\rangle \langle {\sigma}\_{i}^{{22}}\rangle + \langle a\rangle \langle {\sigma}\_{i}^{{22}} {\sigma}\_{j}^{{22}}\rangle -2 \langle a\rangle \langle {\sigma}\_{i}^{{22}}\rangle \langle {\sigma}\_{j}^{{22}}\rangle \right) {g}\_{i} \\
\frac{d}{dt} \langle {\sigma}\_{i}^{{22}} {\sigma}\_{j}^{{21}}\rangle =& 1 i \langle a^\dagger {\sigma}\_{i}^{{22}}\rangle {g}\_{j} + \frac{1}{2} i \langle {\sigma}\_{i}^{{12}} {\sigma}\_{j}^{{21}}\rangle \Omega + \frac{-1}{2} i \langle {\sigma}\_{i}^{{21}} {\sigma}\_{j}^{{21}}\rangle \Omega -1.5 \langle {\sigma}\_{i}^{{22}} {\sigma}\_{j}^{{21}}\rangle \gamma -1 i \langle {\sigma}\_{i}^{{22}} {\sigma}\_{j}^{{21}}\rangle {\delta}a -1 i \langle {\sigma}\_{i}^{{22}} {\sigma}\_{j}^{{22}}\rangle \Omega + \frac{1}{2} i \langle {\sigma}\_{i}^{{22}}\rangle \Omega + 1 i \left( \langle a^\dagger {\sigma}\_{i}^{{12}}\rangle \langle {\sigma}\_{j}^{{21}}\rangle + \langle a^\dagger {\sigma}\_{j}^{{21}}\rangle \langle {\sigma}\_{i}^{{12}}\rangle + \langle a^\dagger\rangle \langle {\sigma}\_{i}^{{12}} {\sigma}\_{j}^{{21}}\rangle -2 \langle a^\dagger\rangle \langle {\sigma}\_{i}^{{12}}\rangle \langle {\sigma}\_{j}^{{21}}\rangle \right) {g}\_{i} -2 i \left( \langle a^\dagger {\sigma}\_{i}^{{22}}\rangle \langle {\sigma}\_{j}^{{22}}\rangle + \langle a^\dagger {\sigma}\_{j}^{{22}}\rangle \langle {\sigma}\_{i}^{{22}}\rangle + \langle a^\dagger\rangle \langle {\sigma}\_{i}^{{22}} {\sigma}\_{j}^{{22}}\rangle -2 \langle a^\dagger\rangle \langle {\sigma}\_{i}^{{22}}\rangle \langle {\sigma}\_{j}^{{22}}\rangle \right) {g}\_{j} -1 i \left( \langle a {\sigma}\_{i}^{{21}}\rangle \langle {\sigma}\_{j}^{{21}}\rangle + \langle a {\sigma}\_{j}^{{21}}\rangle \langle {\sigma}\_{i}^{{21}}\rangle + \langle a\rangle \langle {\sigma}\_{i}^{{21}} {\sigma}\_{j}^{{21}}\rangle -2 \langle a\rangle \langle {\sigma}\_{i}^{{21}}\rangle \langle {\sigma}\_{j}^{{21}}\rangle \right) {g}\_{i} \\
\frac{d}{dt} \langle {\sigma}\_{i}^{{12}} {\sigma}\_{j}^{{12}}\rangle =& -1 i \langle a {\sigma}\_{i}^{{12}}\rangle {g}\_{j} -1 i \langle a {\sigma}\_{j}^{{12}}\rangle {g}\_{i} -1.0 \langle {\sigma}\_{i}^{{12}} {\sigma}\_{j}^{{12}}\rangle \gamma + 2 i \langle {\sigma}\_{i}^{{12}} {\sigma}\_{j}^{{12}}\rangle {\delta}a + 1 i \langle {\sigma}\_{i}^{{12}} {\sigma}\_{j}^{{22}}\rangle \Omega + \frac{-1}{2} i \langle {\sigma}\_{i}^{{12}}\rangle \Omega + \frac{-1}{2} i \langle {\sigma}\_{j}^{{12}}\rangle \Omega + 1 i \langle {\sigma}\_{i}^{{22}} {\sigma}\_{j}^{{12}}\rangle \Omega + 2 i \left( \langle a {\sigma}\_{i}^{{12}}\rangle \langle {\sigma}\_{j}^{{22}}\rangle + \langle a {\sigma}\_{j}^{{22}}\rangle \langle {\sigma}\_{i}^{{12}}\rangle + \langle a\rangle \langle {\sigma}\_{i}^{{12}} {\sigma}\_{j}^{{22}}\rangle -2 \langle a\rangle \langle {\sigma}\_{i}^{{12}}\rangle \langle {\sigma}\_{j}^{{22}}\rangle \right) {g}\_{j} + 2 i \left( \langle a {\sigma}\_{j}^{{12}}\rangle \langle {\sigma}\_{i}^{{22}}\rangle + \langle a {\sigma}\_{i}^{{22}}\rangle \langle {\sigma}\_{j}^{{12}}\rangle + \langle a\rangle \langle {\sigma}\_{i}^{{22}} {\sigma}\_{j}^{{12}}\rangle -2 \langle a\rangle \langle {\sigma}\_{j}^{{12}}\rangle \langle {\sigma}\_{i}^{{22}}\rangle \right) {g}\_{i} \\
\frac{d}{dt} \langle {\sigma}\_{i}^{{22}} {\sigma}\_{j}^{{22}}\rangle =& \frac{1}{2} i \langle {\sigma}\_{i}^{{12}} {\sigma}\_{j}^{{22}}\rangle \Omega + \frac{-1}{2} i \langle {\sigma}\_{i}^{{21}} {\sigma}\_{j}^{{22}}\rangle \Omega + \frac{1}{2} i \langle {\sigma}\_{i}^{{22}} {\sigma}\_{j}^{{12}}\rangle \Omega + \frac{-1}{2} i \langle {\sigma}\_{i}^{{22}} {\sigma}\_{j}^{{21}}\rangle \Omega -2.0 \langle {\sigma}\_{i}^{{22}} {\sigma}\_{j}^{{22}}\rangle \gamma + 1 i \left( \langle a^\dagger {\sigma}\_{i}^{{12}}\rangle \langle {\sigma}\_{j}^{{22}}\rangle + \langle a^\dagger {\sigma}\_{j}^{{22}}\rangle \langle {\sigma}\_{i}^{{12}}\rangle + \langle a^\dagger\rangle \langle {\sigma}\_{i}^{{12}} {\sigma}\_{j}^{{22}}\rangle -2 \langle a^\dagger\rangle \langle {\sigma}\_{i}^{{12}}\rangle \langle {\sigma}\_{j}^{{22}}\rangle \right) {g}\_{i} + 1 i \left( \langle a^\dagger {\sigma}\_{j}^{{12}}\rangle \langle {\sigma}\_{i}^{{22}}\rangle + \langle a^\dagger {\sigma}\_{i}^{{22}}\rangle \langle {\sigma}\_{j}^{{12}}\rangle + \langle a^\dagger\rangle \langle {\sigma}\_{i}^{{22}} {\sigma}\_{j}^{{12}}\rangle -2 \langle a^\dagger\rangle \langle {\sigma}\_{j}^{{12}}\rangle \langle {\sigma}\_{i}^{{22}}\rangle \right) {g}\_{j} -1 i \left( \langle a {\sigma}\_{i}^{{21}}\rangle \langle {\sigma}\_{j}^{{22}}\rangle + \langle a {\sigma}\_{j}^{{22}}\rangle \langle {\sigma}\_{i}^{{21}}\rangle + \langle a\rangle \langle {\sigma}\_{i}^{{21}} {\sigma}\_{j}^{{22}}\rangle -2 \langle a\rangle \langle {\sigma}\_{i}^{{21}}\rangle \langle {\sigma}\_{j}^{{22}}\rangle \right) {g}\_{i} -1 i \left( \langle a {\sigma}\_{j}^{{21}}\rangle \langle {\sigma}\_{i}^{{22}}\rangle + \langle a {\sigma}\_{i}^{{22}}\rangle \langle {\sigma}\_{j}^{{21}}\rangle + \langle a\rangle \langle {\sigma}\_{i}^{{22}} {\sigma}\_{j}^{{21}}\rangle -2 \langle a\rangle \langle {\sigma}\_{j}^{{21}}\rangle \langle {\sigma}\_{i}^{{22}}\rangle \right) {g}\_{j}
\end{align}
