# Supplementary material for: Collectively enhanced Ramsey readout by cavity sub- to superradiant transition
Source: Nat Commun. 2024 Feb 5;15:1084. doi: 10.1038/s41467-024-45420-x (PMC10844618; doi:10.1038/s41467-024-45420-x)
Supplement: Supplementary file 5 — Supplementary Code 2 [file 41467_2024_45420_MOESM5_ESM.html]

cavity\_sub-to-superradiance\_simulations


# Cavity Sub- to Superradiance - Simulations¶

In [1]:

```
# Julia-1.9.1
using QuantumCumulants #v0.2.23
using OrdinaryDiffEq #v6.44.1
using ModelingToolkit #v8.70.0
using PyPlot #v2.11.2
using ProgressMeter #v1.9.0
```

In [2]:

```
M = 8 # number of cluster (sample different positions)

# Hilbert space
hf = FockSpace(:cavity) 
ha(i) = NLevelSpace(Symbol(:atom,i),2)
h = tensor(hf, [ha(i) for i=1:M]...)

# Fundamental operators
a = Destroy(h,:a,1) 
σ(i,j,k) = IndexedOperator(Transition(h,Symbol(:σ_,k,:_),i,j,k.aon), k)

# Parameters
@cnumbers γ κ δc δa Nc
g(ind) = cnumber(Symbol(:g,ind))

@syms t::Real #time variable
@register_symbolic Ω(t)

# Summation indices
i(ind) = Index(h,Symbol(:i,ind),Nc,ha(ind))
j(ind) = Index(h,Symbol(:j,ind),Nc,ha(ind))
k(ind) = Index(h,Symbol(:k,ind),Nc,ha(ind))
extra_indices = [[i(ind) for ind=1:M]; [j(ind) for ind=1:M]]

# Symbolic list of paramters
ps = [γ, κ, δc, δa, Nc, [g(i) for i=1:M]...];
```

In [3]:

```
# Hamiltonian
H_0 = -δc*a'a - δa*sum( ∑(σ(2,2,k(ind)),k(ind)) for ind=1:M)
H_int = sum( g(ind)*(a'∑(σ(1,2,k(ind)),k(ind)) + a*∑(σ(2,1,k(ind)),k(ind))) for ind=1:M)
H_laser = Ω(t)*sum( ( ∑(σ(1,2,k(ind)),k(ind)) + ∑(σ(2,1,k(ind)),k(ind))) for ind=1:M)
H = H_0 + H_int + H_laser

# Jump operators & rates
J = [a, [σ(1,2,k(ind)) for ind=1:M]...] 
R = [κ, [γ for ind=1:M]...];
```

In [4]:

```
eqs = meanfield([a'a], H, J; rates=R, order=2)
eqs_c = complete(eqs; extra_indices)
eqs_sc = scale(eqs_c)
@named sys = ODESystem(eqs_sc)
println("Number of eqs. = $(length(eqs_sc))")
```

```
Number of eqs. = 215
```

In [5]:

```
# Numerical parameters
γ_ = 2π*7.5
κ_ = 2π*780
δc_ = 0.0
δa_ = 0.0
Ω_ = 2π*833
g_sin = [sin((j+1/2)*π/2/(M/2)) for j=0:Int(M/2)-1]
g_ = 2π*0.610*[g_sin; -g_sin]

# Threshold scan parameters
Nc_t = 2e7/M
T_end_t = 0.01
# Ramsey scan parameters
Nc_r = 4e7/M

# Numeric list of parameters
p0_t = [γ_, κ_, δc_, δa_, Nc_t, g_...]
p0_r = [γ_, κ_, δc_, δa_, Nc_r, g_...]

# initial state
u0 = zeros(ComplexF64, length(eqs_sc));
```

## Time evolution¶

In [6]:

```
tΩ = π/Ω_*3/4
Ω(t) = Ω_/2* (t<tΩ)
prob = ODEProblem(sys,u0,(0.0, T_end_t), ps.=>p0_t)
sol = solve(prob, Tsit5(); reltol=1e-8, abstol=1e-8, maxiters=1e7);
```

In [7]:

```
figure("n(t)")
plot(sol.t, real.(sol[a'a]))
xlabel("t [ms]")
ylabel("⟨a⁺a⟩");
```

## Threshold scan¶

In [8]:

```
tΩ_ls = [0.4:0.05:1;]*π/Ω_ # reduced resolution
l_tΩ = length(tΩ_ls)

t_peak_ls_t = zeros(l_tΩ)
n_peak_ls_t = zeros(l_tΩ)
n_out_ls_t = zeros(l_tΩ)

# this can take a couple of minutes
prog_t = Progress(l_tΩ)
for it=1:l_tΩ 
    tΩ = tΩ_ls[it]
    Ω(t) = Ω_/2*(t<tΩ)
    prob_t = ODEProblem(sys,u0,(0.0, T_end_t), ps.=>p0_t) 
    sol_t = solve(prob_t, Tsit5(); reltol=1e-8, abstol=1e-8, maxiters=1e7)
    T_t = sol_t.t
    n_t = real.(sol_t[a'a])
    n_p_t, T_p_t = findmax(n_t)
    
    t_peak_ls_t[it] = T_t[T_p_t] - tΩ
    n_peak_ls_t[it] = n_p_t
    n_out_ls_t[it] = sum((T_t[it+1]-T_t[it])*(n_t[it+1]+n_t[it])/2 for it=1:length(n_t)-1)*κ_
    next!(prog_t)
end
```

```
Progress: 100%|█████████████████████████████████████████| Time: 0:01:47
```

In [9]:

```
s22(tΩ) = (sin(Ω_*tΩ/2))^2
n_out_ls_t_ = n_out_ls_t ./ maximum(n_out_ls_t)
n_peak_ls_t_ = n_peak_ls_t ./ maximum(n_peak_ls_t)

figure("Threshold", figsize=[8,4])
subplot(121)
plot(s22.(tΩ_ls), n_out_ls_t_, label="total output")
plot(s22.(tΩ_ls), n_peak_ls_t_, label="peak")
xlabel("sin²(Ω tp/2)")
ylabel("photon number (normalized)")
legend(loc="lower right")
subplot(122)
plot(s22.(tΩ_ls), t_peak_ls_t*1e3)
xlabel("sin²(Ω tp/2)")
ylabel("pulse peak delay time [μs]")
xlim(0.6)
tight_layout()
;
```

## Ramsey scan¶

In [10]:

```
tΩ = 300e-9*1e3 # pulse time 300ns (in units of kHz)
tf = 5e-6*1e3 - tΩ # free evolution time 500μs (end to end)
Ω(t) = t % (tΩ+tf) < tΩ ? Ω_/2 : 0.0 # Ramsey sequence

δ_ls = [-160:5:160;]*2π # reduced resolution
l_δ = length(δ_ls)

n_peak_ls_r = zeros(l_δ)

prob_r = ODEProblem(sys,u0,(0.0, 2tf), ps.=>p0_r)
p_sys = parameters(sys)
p_idx = [findfirst(isequal(p), ps) for p∈p_sys]

# this can take a couple of minutes
prog_r = Progress(l_δ)
Threads.@threads for it=1:l_δ
    δ_ = δ_ls[it] # δc=δa
    p0_r_ = [γ_, κ_, δ_, δ_, Nc_r, g_...]
    prob_r_ = remake(prob_r, p=p0_r_[p_idx])
    sol_r = solve(prob_r_, Tsit5(); reltol=1e-8, abstol=1e-8, maxiters=1e7)
    T_r = sol_r.t
    n_r = real.(sol_r[a'a])
    n_p_r, T_p_r = findmax(n_r)

    n_peak_ls_r[it] = n_p_r
    next!(prog_r)
end
```

```
Progress: 100%|█████████████████████████████████████████| Time: 0:00:53
```

In [11]:

```
n_peak_ls_r_ = n_peak_ls_r ./ maximum(n_peak_ls_r)

figure("Ramsey fringes")
plot(δ_ls/2π, n_peak_ls_r_)
xlabel("δ/2π [kHz]")
ylabel("peak photon number (normalized)");
```
